# Supplementary material for: Slap restricts oncogenic Src-family kinase signaling to maintain colonic epithelial homeostasis
Source: eLife. 2026 Sep 8;15:RP110324. doi: 10.7554/eLife.110324 (PMC13553065; doi:10.7554/eLife.110324)
Supplement: Supplementary file 3 [file elife-110324-fig4-data1.pdf]

Fig. 4A

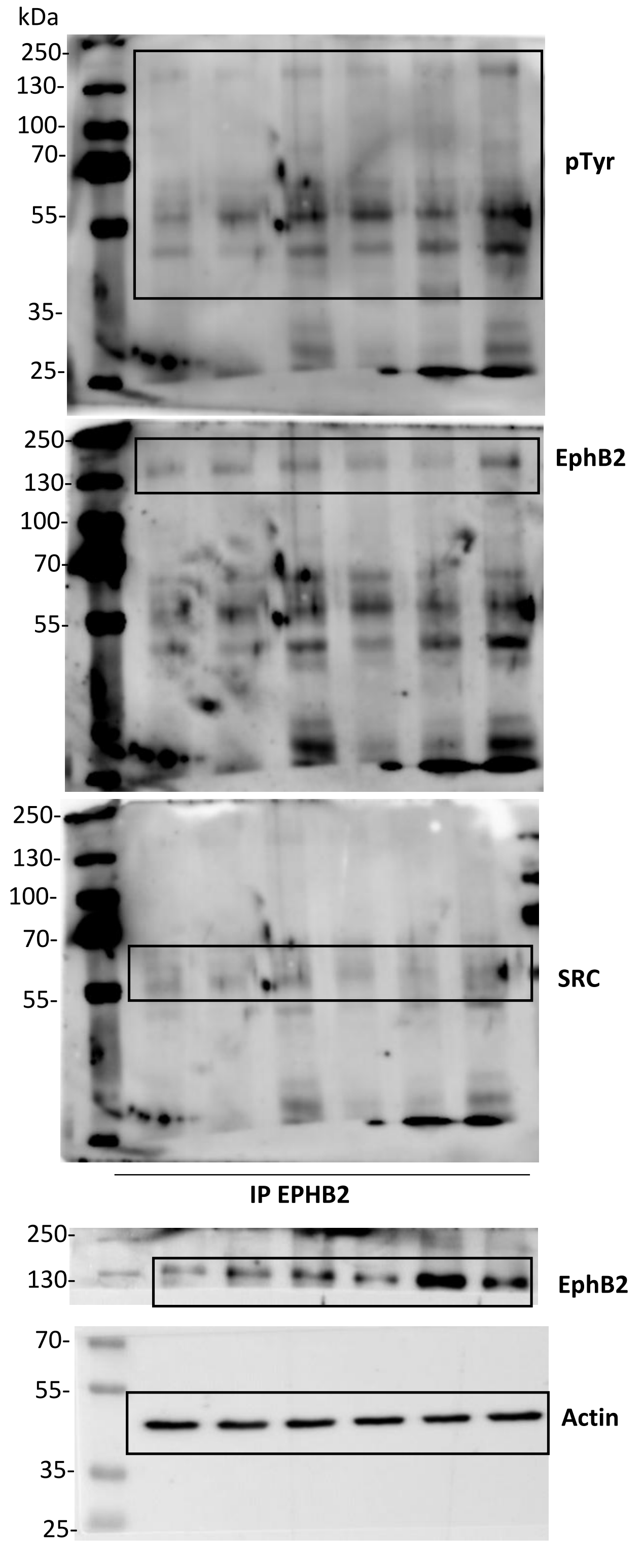

Figure 4, Source Data 1. Original membranes corresponding to Figure 4A. Specific lanes presented in the final manuscript are indicated with black box.

Fig. 4C

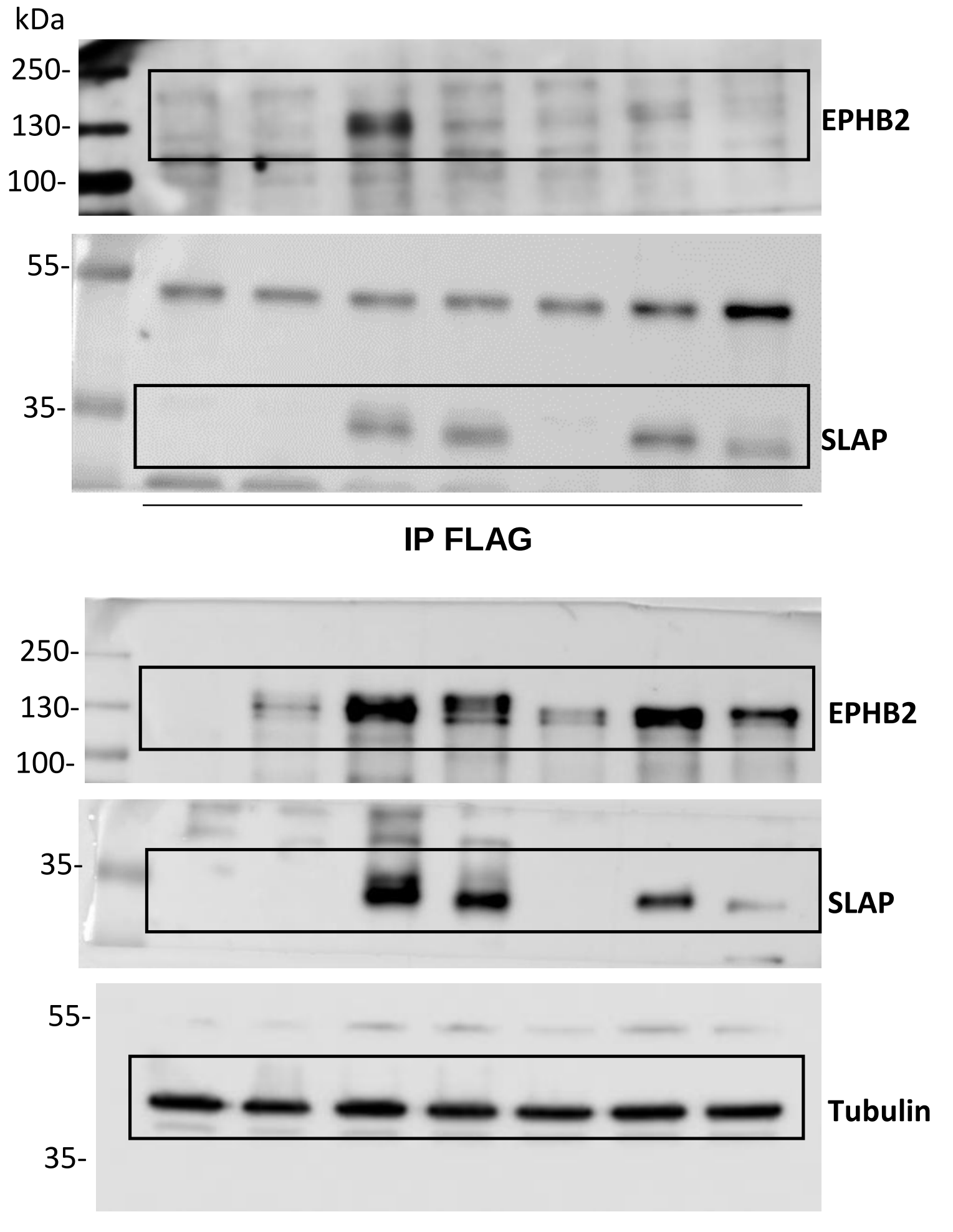

Figure 4, Source Data 1. Original membranes corresponding to Figure 4C. Specific lanes presented in the final manuscript are indicated with black box.
