# Supplementary material for: Slap restricts oncogenic Src-family kinase signaling to maintain colonic epithelial homeostasis
Source: eLife. 2026 Sep 8;15:RP110324. doi: 10.7554/eLife.110324 (PMC13553065; doi:10.7554/eLife.110324)
Supplement: Supplementary file 4 [file elife-110324-fig4-figsupp1-data1.pdf]

Sup. Fig. 4A

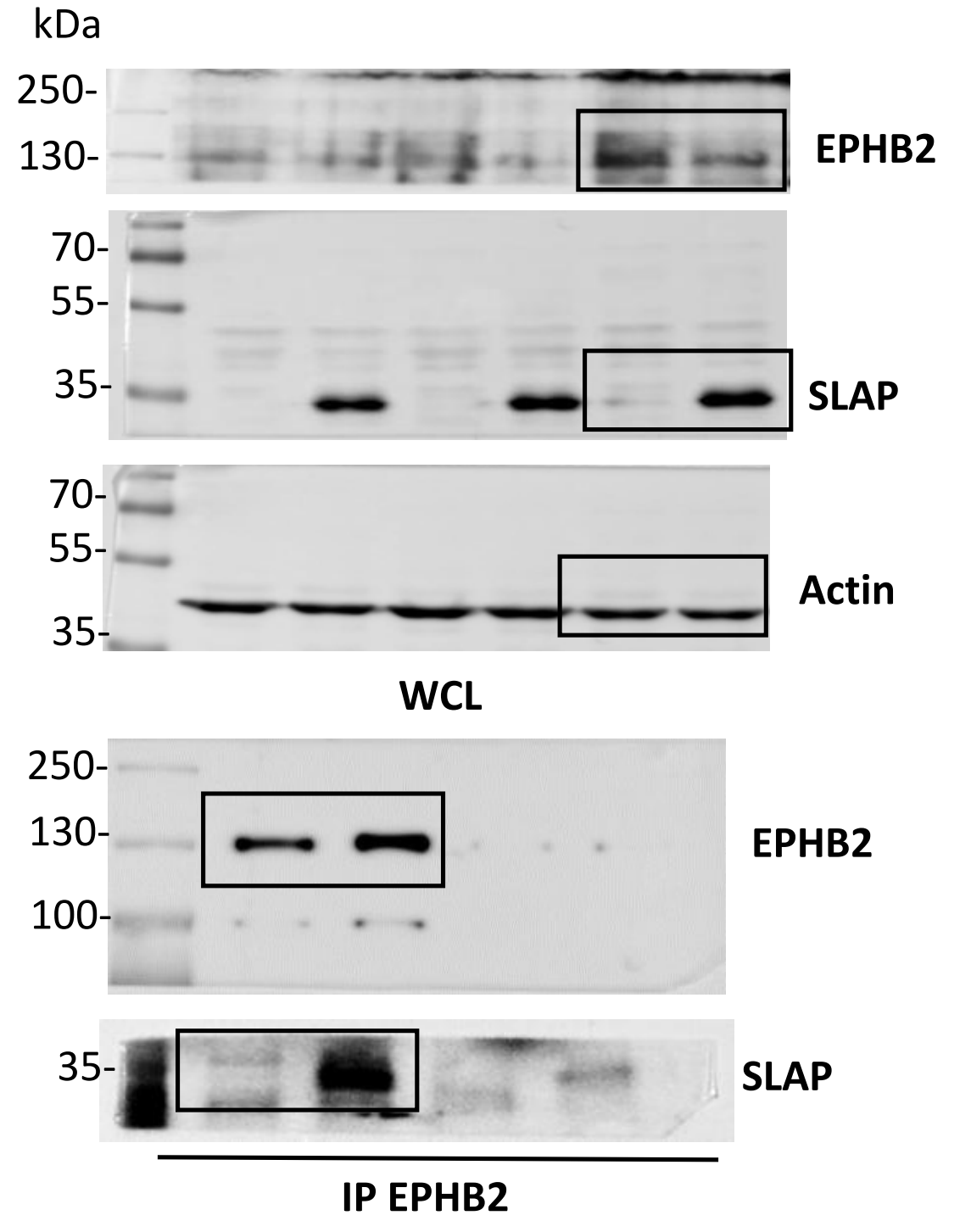

Sup. Fig. 4B

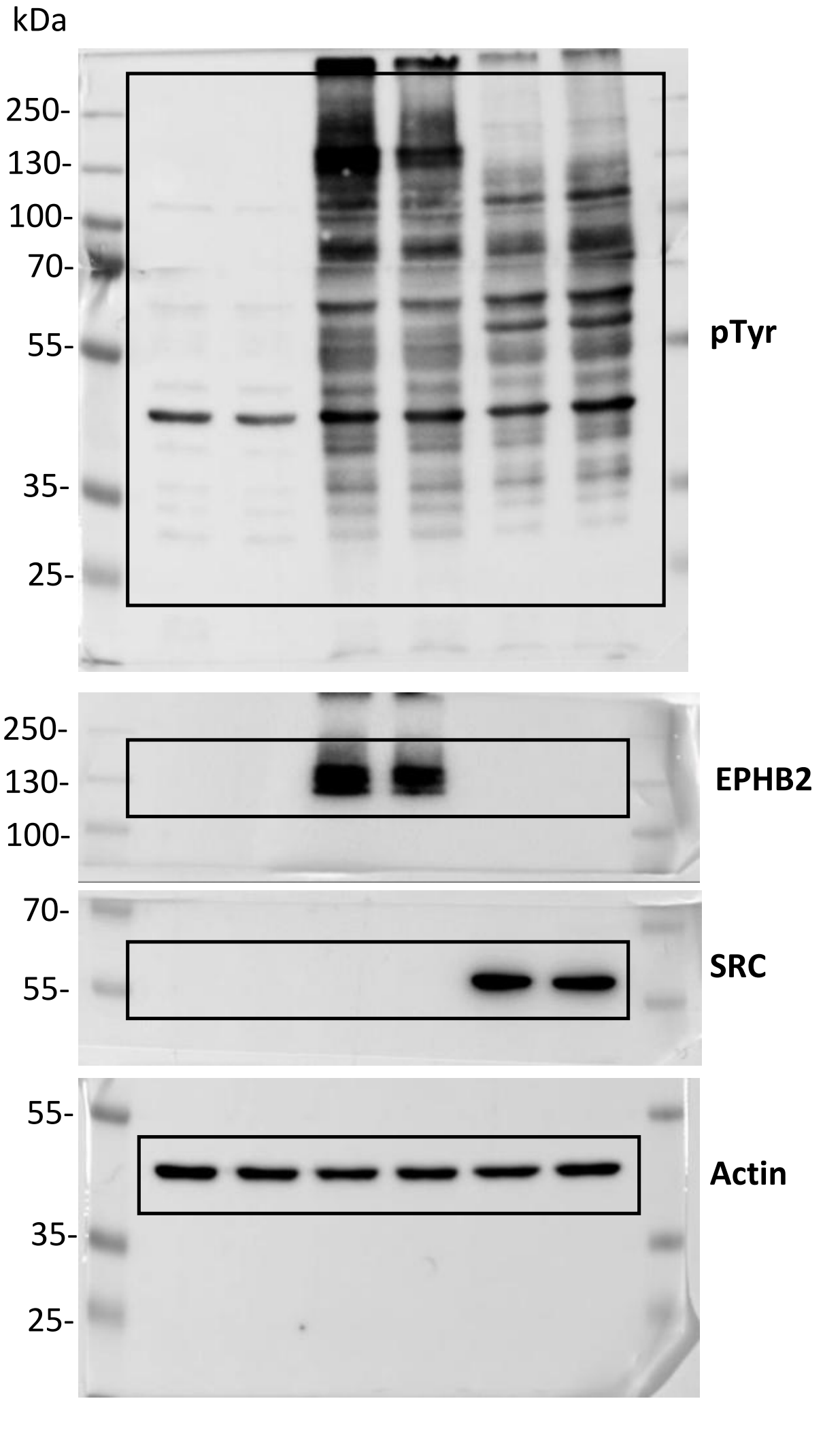

Supplement Figure 4, Source Data 1. Original membranes corresponding to Sup. Fig. 4A and 4B. Specific lanes presented in the final manuscript are indicated with black box.
