# Supplementary material for: Slap restricts oncogenic Src-family kinase signaling to maintain colonic epithelial homeostasis
Source: eLife. 2026 Sep 8;15:RP110324. doi: 10.7554/eLife.110324 (PMC13553065; doi:10.7554/eLife.110324)
Supplement: Supplementary file 5 [file elife-110324-fig5-data1.pdf]

Fig. 5A

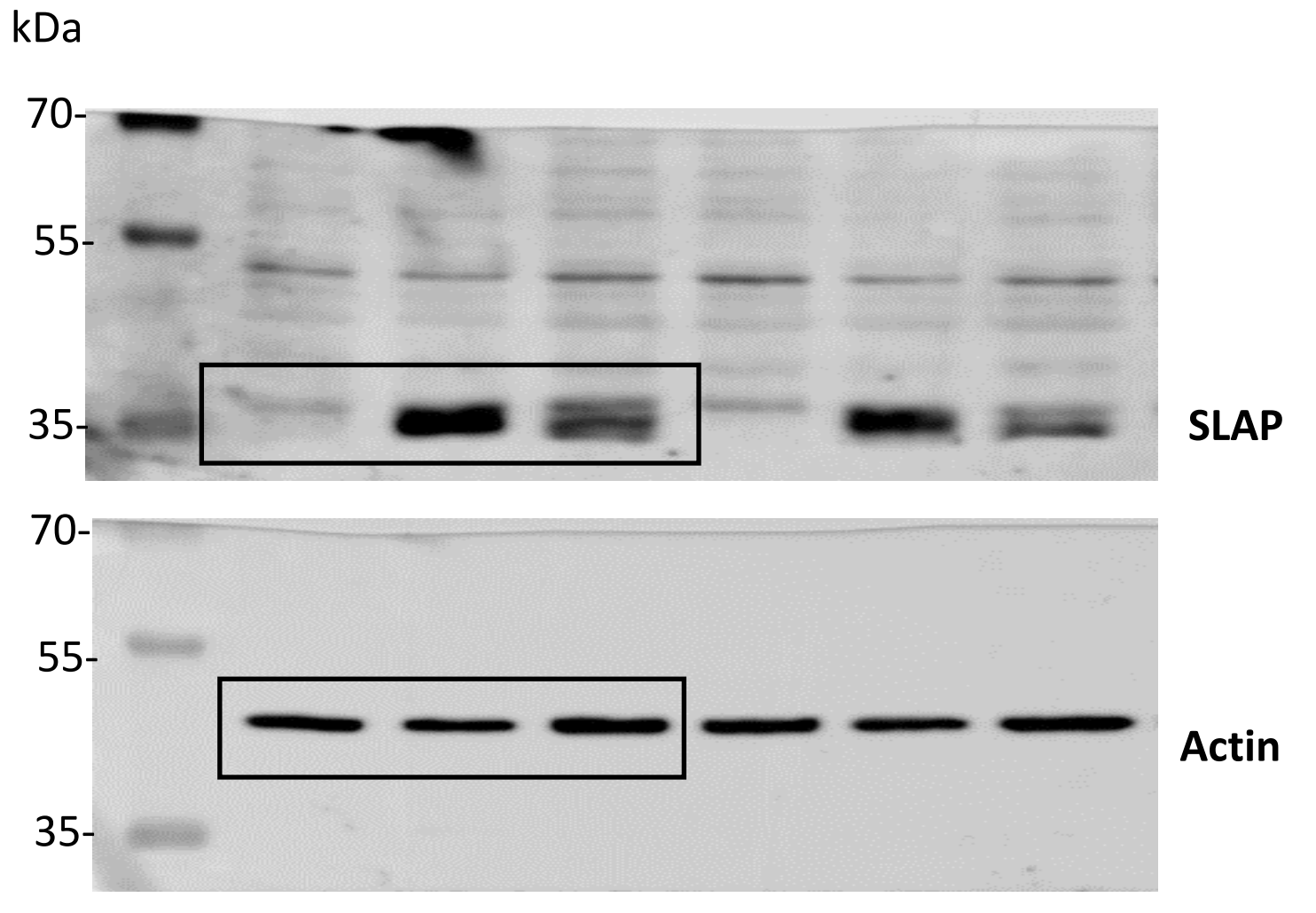

Figure 5, Source Data 1. Original membranes corresponding to Figure 5A. Specific lanes presented in the final manuscript are indicated with black box.

Sup. Fig. 4A

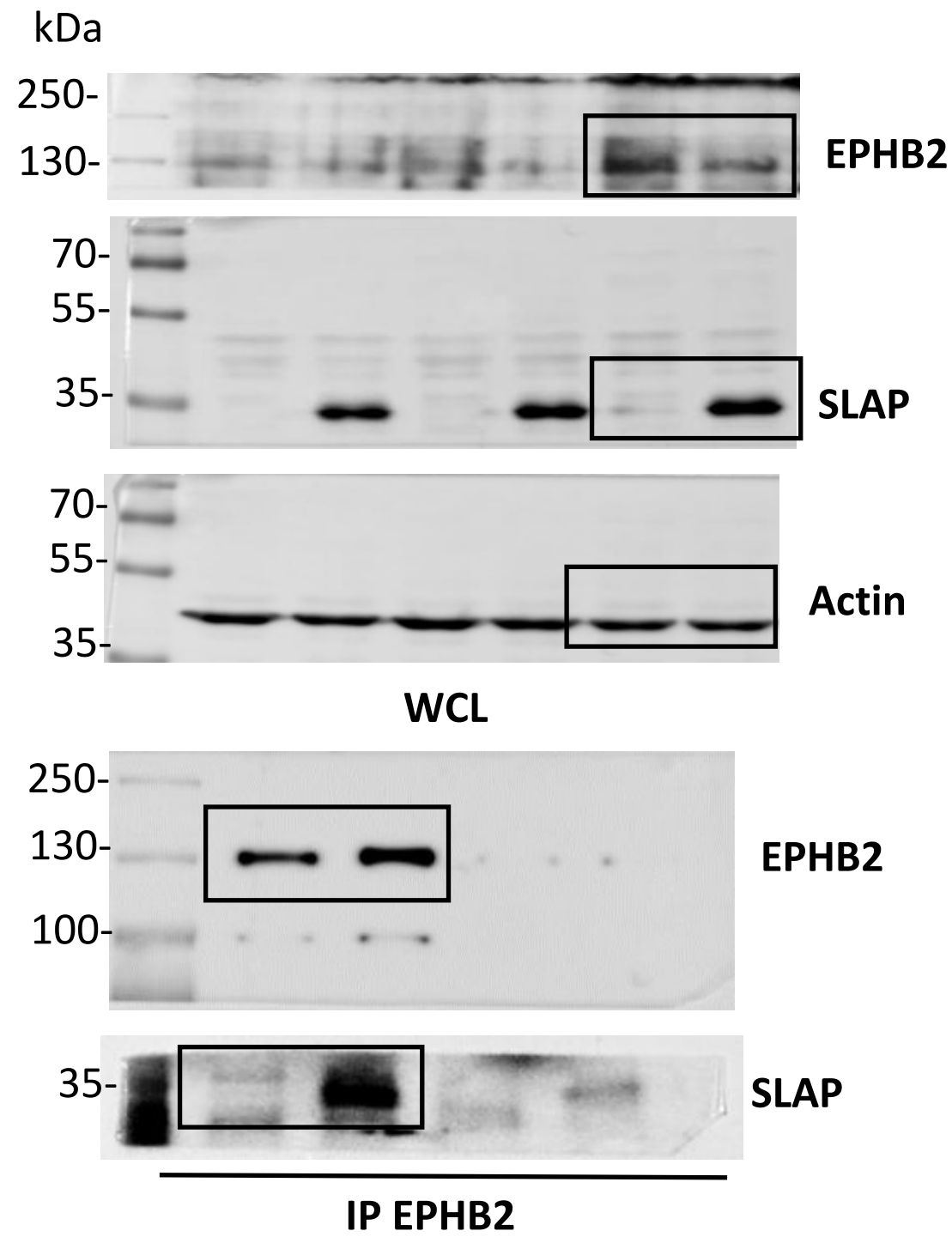

Sup. Fig. 4B

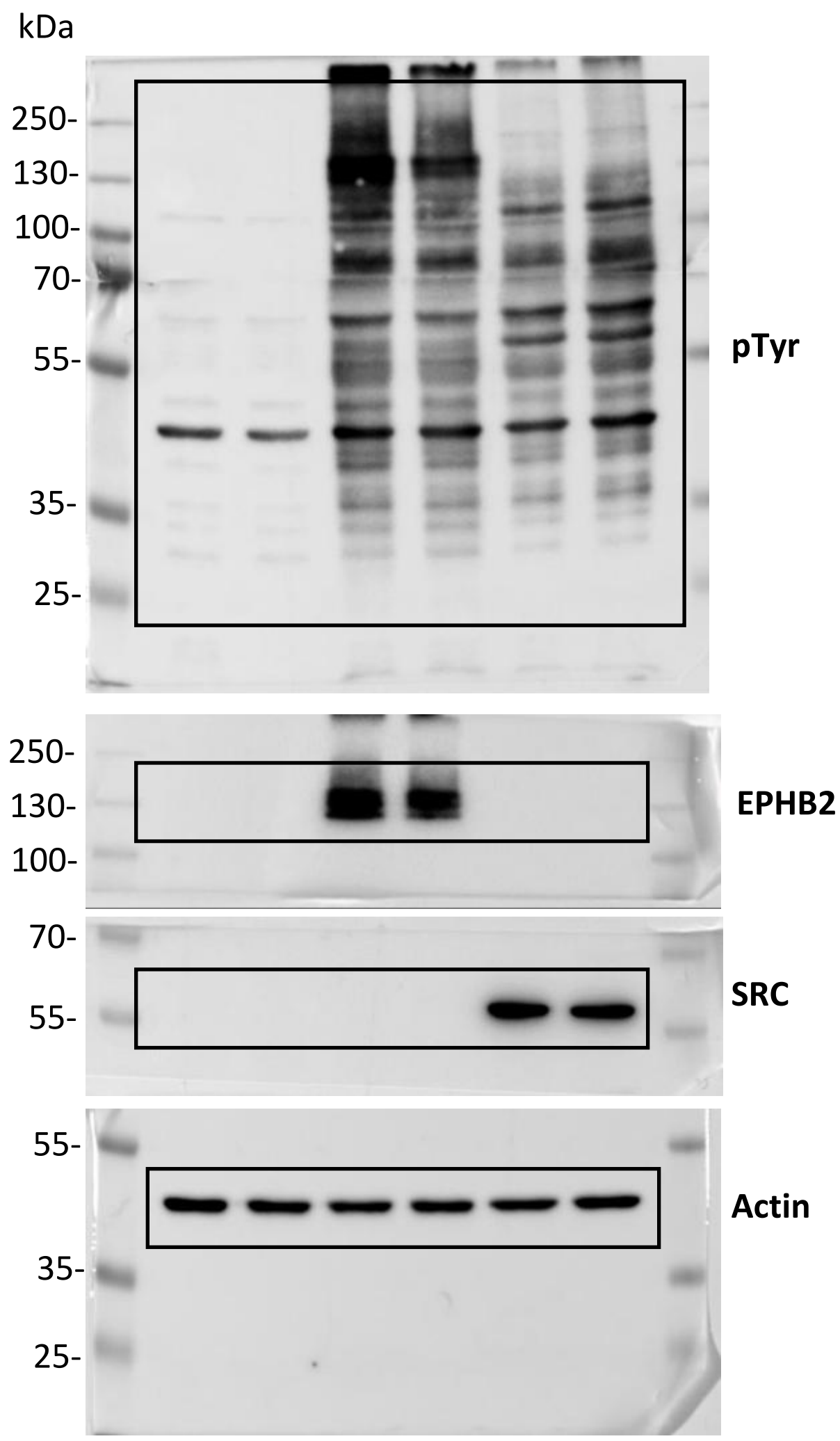

Supplement Figure 4, Source Data 1. Original membranes corresponding to Sup. Fig. 4A and 4B. Specific lanes presented in the final manuscript are indicated with black box.
