# Supplementary material for: Slap restricts oncogenic Src-family kinase signaling to maintain colonic epithelial homeostasis
Source: eLife. 2026 Sep 8;15:RP110324. doi: 10.7554/eLife.110324 (PMC13553065; doi:10.7554/eLife.110324)
Supplement: Peer review history

# Editor report: eLife Assessment

**DOI:** 10.7554/eLife.110324.3.sa0

**Authors:** Frederic A Bard

**Affiliations:** Centre de Recherche en Cancérologie de Marseille France

**Keywords:** Important, Solid

This is an **important** study on the role of Slap in restricting Src activity and proliferation of colonic cells in vivo. The authors present **solid** evidence that an EPHB2-SRC signaling axis stimulates the proliferation of colon precursors and is controlled by the Src-binding protein SLAP, whose loss promotes tumorigenesis.

# Referee report: Reviewer #1 (Public review)

**DOI:** 10.7554/eLife.110324.3.sa1

Naim et al., use genetically engineered mouse models and tissue culture cell lines to investigate the role of the SLAP adaptor protein in colonic epithelium and colon tumour formation. The SLAP adaptor protein is known to be a negative regulator of tyrosine kinase signaling in hematopoietic cells but its role outside the immune system is less well defined. Here the authors use genetically engineered SLAP deficient mice, tissue specific SLAP KO, and colonic organoids to demonstrate that SLAP is expressed in cells of the colonic epithelium where it acts as a cell autonomous regulator of proliferation and differentiation. In addition, they provide biochemical evidence that loss of SLAP expression in cultured colonic organoids results in increased Src family kinase activity and global tyrosine phosphorylation, consistent with its known role as a suppressor of tyrosine kinase activity in immune cells. Consistently, treatment with a SRC kinase inhibitor inhibited growth of SLAP deficient organoids. These data provide solid evidence of a cell autonomous role of SLAP in the colonic epithelium.

Using a chemically induced model of colitis-associated cancer the authors demonstrate that inactivation of SLAP shows a trend toward increased tumor formation as well as significantly increased Src family kinase activity within tumors. Tumor spheres from SLAP deficient animals showed enhanced growth that was suppressed by treatment with a Src family kinase inhibitor. Of note, the latter effect was specific to SLAP deficient tumor spheres. These observations are convincing and support the authors conclusion that SLAP has a tumor suppressor role in CRC through inhibition of SFK signaling.

Mechanistically, elevated expression of the EPHB2 receptor tyrosine kinase was detected in immunoblots and by IHC of SLAP KO colonic crypts. In addition, in SLAP deficient crypts, levels of phosphorylated EPHB2 are increased and associated with activated SRC family kinases. Using an EPHB2 inhibitor, the role of EPHB2 in the growth of SLAP deficient colonic organoids, and downstream SRC phosphorylation was demonstrated. The authors also show that low expression of SLAP in human CRC cell line organoids sensitizes to the growth inhibitory effects EPH inhibition which can be reversed by SLAP over expression but not expression of a SH2/SH3 mutant form of SLAP.

Overall, this work provides evidence of SLAP adaptor function in restricting EPH tyrosine kinase signaling the colonic epithelium and suggests that loss of SLAP expression promotes tumorigenesis in this context.

# Referee report: Reviewer #2 (Public review)

**DOI:** 10.7554/eLife.110324.3.sa2

Summary:

Protein tyrosine kinases are submitted to diverse regulatory mechanisms controling their activity in normal situation. The authors previously identified SLAP (Src-like adaptor protein), a negative regulator of receptor tyrosine kinase (RTK) signaling, as a key suppressor of the cytoplasmic tyrosine kinase SRC in the normal colon and demonstrated that SLAP is downregulated in a majority of colorectal cancers (CRCs).

In this study, the authors further explored slap functions in mouse models using constitutive and inducible epithelial-specific Slap deletion (villin-CreERT2 model). They found that loss of slap augments colonic epithelial cell proliferation and that induction of tumorigenesis by the AOM/DSS protocol mimicking CRC leads to more aggressive tumors in the absence of slap. This effect is apparently cell-autonomous as growth of normal and tumoral colonic organoids is SLAP-dependent in in vitro settings. Finally, the authors define that, in colon, SLAP represses EphB2, an RTK lying upstream of SRC, and show that inhibitors of EphB2 can partially limit tumorigenic development in vitro.

Strengths:

The manuscript is clearly and concisely written, making it easy to follow. Data obtained in the mouse models are very convincing.

Weaknesses:

Direct evidence that EphB2 is activated/phosphorylated in the absence of SLAP is lacking as conclusions are only based on results obtained with inhibitors. Some other issues have to be addressed before acceptance, in particular the relevance of the findings in CRC patients.

Comments on revised version.

The authors have satisfactorily addressed my concerns.

# Author comment: Author response

**DOI:** 10.7554/eLife.110324.3.sa3

**Authors:** Dana Naim, Zouheir Houhou, Florent Cauchois, Kevin Espie, Valerie Simon, Yvan Boublik, Francina Langa, Zeinab Homayed, Conception Paul, Morgan Maillard, Michael Hahne, Julie Pannequin, Julie Nguyen, Audrey Sirvent, Serge Roche

**Affiliations:** Centre de Recherche en Biologie cellulaire de Montpellier montpellier France; Centre de Recherche en Biologie cellulaire de Montpellier montpellier France; Centre de Recherche en Biologie cellulaire de Montpellier montpellier France; Centre de Recherche en Biologie cellulaire de Montpellier montpellier France; Centre de Recherche en Biologie cellulaire de Montpellier Montpellier France; Centre de Recherche en Biologie cellulaire de Montpellier montpellier France; Institut Pasteur Paris France; Institute of Functional Genomics (IGF) Montpellier France; Institut de Génétique Moléculaire de Montpellier Montpellier France; Institut de Recherche en Cancérologie de Montpellier montpellier France; Institut de Génétique Moléculaire de Montpellier Montpellier France; Institute of Functional Genomics (IGF) Montpellier France; Institute of Functional Genomics (IGF) Montpellier France; Centre de Recherche en Biologie cellulaire de Montpellier Montpellier France; Centre de Recherche en Biologie cellulaire de Montpellier Montpellier France

The following is the authors’ response to the original reviews.

**Public Reviews:**

**Reviewer #1 (Public review):**

Naim et al. use genetically engineered mouse models and tissue culture cell lines to investigate the role of the SLAP adaptor protein in colonic epithelium and colon tumour formation. The SLAP adaptor protein is known to be a negative regulator of tyrosine kinase signaling in hematopoietic cells, but its role outside the immune system is less well defined. Here, the authors use genetically engineered SLAP-deficient mice, tissue-specific SLAP KO, and colonic organoids to demonstrate that SLAP is expressed in cells of the colonic epithelium, where it acts as a cell-autonomous regulator of proliferation and differentiation. In addition, they provide biochemical evidence that loss of SLAP expression in cultured colonic organoids results in increased Src family kinase activity and global tyrosine phosphorylation, consistent with its known role as a suppressor of tyrosine kinase activity in immune cells. Consistently, treatment with an SRC kinase inhibitor inhibited the growth of SLAP-deficient organoids. These data provide solid evidence of a cell-autonomous role of SLAP in the colonic epithelium.

This work would be improved by further description and interpretation of the SLAP expression pattern shown in the constitutive and tissue-specific KO to further support the conclusions made. In Supplementary Figure 1, magnification of the colon epithelium areas with SLAP expression shown by b-gal and anti-SLAP staining, highlighting regions of interest, would better support the conclusions regarding SLAP expression in specific regions of the colon epithelium. In Supplementary Figure 1B, the authors should indicate that the SLAP staining referred to is epithelial and in resident immune cells, as is mentioned in the text. Also, magnification of the boxed area of LRG5 staining in Figure 1 would improve this figure.

We thank the reviewer for their positive and constructive evaluation of our work.

We have revised Fig 1 and S1 to better highlight SLAP expression patterns. Specifically, we have included higher-magnification images of the colonic epithelial regions, with clearly indicated regions of interest (new Figure S1). We have also clarified in the legend that SLAP staining is observed in both epithelial and resident immune cells, as described in the text. Additionally, we have provided a magnified view of the boxed area showing LGR5 staining in Figure 1 to improve clarity.

Using a chemically induced model of colitis-associated cancer, the authors demonstrate that inactivation of SLAP shows a trend toward increased tumor formation (though this did not reach significance) as well as increased Src family kinase activity within tumors. Tumor spheres from SLAP-deficient animals showed enhanced growth that was suppressed by treatment with a Src family kinase inhibitor. Of note, the latter effect was specific to SLAP-deficient tumor spheres. These observations are convincing and support the authors' conclusion that SLAP has a tumor suppressor role in CRC through inhibition of SFK signaling.

Mechanistically, elevated expression of the RTK, EphB2, was detected in immunoblots of SLAP KO colonic crypts, while overexpression of SLAP in CRC cell lines downregulated EphB2 protein levels. Using an EPHB2 inhibitor, the role of EPHB2 in the growth of SLAP-deficient colonic organoids was demonstrated. While these data generally support the authors' conclusion that SLAP limits colonic organoid growth by downregulating RTKS such as EphB2 and downstream Src family kinase activity, they do not show which cell types/regions in the colonic epithelium have increased EPHB2 protein and how this relates to SLAP and phospho-SRC expression, as shown in Figure 1 and Figure S1 immunocytochemistry. The expression of EphB2 and its role in colonic tumorsphere growth were not investigated.

Overall, this work provides evidence of SLAP adaptor function in restricting tyrosine kinase signaling in the colonic epithelium, and suggests that loss of SLAP expression could promote tumorigenesis in this context.

We thank the reviewer for their positive assessment of our tumour studies and for recognizing the evidence supporting a tumor suppressor role for SLAP through inhibition of SFK signaling.

To address the reviewer’s mechanistic concerns, we performed additional experiments that are now included in the revised manuscript. We confirmed that loss of Slap is associated with increased EPHB2 expression in colonic crypts by IHC (new Figure 4B) and directly tested the role of EPHB2 in the Slap-deficient phenotype: EPH inhibition reduced both pTyr levels and SRC activation in Slap-deficient organoids (new Figure S2), demonstrating that SFK hyperactivation depends on upstream EPHB2 signaling. Consistent with this mechanism, we also observed increased EPHB2 tyrosine phosphorylation and active SRC (pSRC) association in isolated colonic epithelial cells following Slap deletion (new Figure 4A).

To extend these findings to the tumour context, we examined the effect of EPH inhibition in human CRC tumoroids (new Figure 5). Pharmacological EPHB2 inhibition reduced tumoroid growth in CRC cells expressing low levels of SLAP, whereas this effect was largely lost upon SLAP overexpression. An SH2- or SH3-inactivating point SLAP mutant failed to suppress tumoroid growth and restored sensitivity to EPHB2 inhibition, further supporting EPHB2 as a critical target of SLAP-mediated tumour suppression. Together, these new data identify EPHB2 as a critical upstream activator of SRC that is negatively regulated by SLAP and strengthen our conclusion that deregulated EPHB2-SRC signaling drives the hyperproliferative phenotype associated with SLAP loss.

**Reviewer #2 (Public review):**

Summary:

Protein tyrosine kinases are subject to diverse regulatory mechanisms controlling their activity in normal situations. The authors previously identified SLAP (Src-like adaptor protein), a negative regulator of receptor tyrosine kinase (RTK) signaling, as a key suppressor of the cytoplasmic tyrosine kinase SRC in the normal colon and demonstrated that SLAP is downregulated in a majority of colorectal cancers (CRCs).

In this study, the authors further explored SLAP functions in mouse models using constitutive and inducible epithelial-specific Slap deletion (villin-CreERT2 model). They found that loss of SLAP augments colonic epithelial cell proliferation and that induction of tumorigenesis by the AOM/DSS protocol mimicking CRC leads to more aggressive tumors in the absence of SLAP. This effect is apparently cell-autonomous as growth of normal and tumoral colonic organoids is SLAP-dependent in in vitro settings. Finally, the authors define that, in colon, SLAP represses EphB2, an RTK lying upstream of SRC, and show that inhibitors of EphB2 can partially limit tumorigenic development in vitro.

Strengths:

The manuscript is clearly and concisely written, making it easy to follow. The data obtained in the mouse models are very convincing.

Weaknesses:

Direct evidence that EphB2 is activated/phosphorylated in the absence of SLAP is lacking, as conclusions are only based on results obtained with inhibitors. Some other issues have to be addressed before acceptance, in particular, the relevance of the findings in CRC patients.

We thank the reviewer for their positive and constructive evaluation of our work.

We agree that direct evidence linking SLAP loss to activation of the EPHB2-SRC pathway would strengthen the study. To address this point, we performed additional experiments that are now included in the revised manuscript. In addition to demonstrating increased EPHB2 expression upon Slap deletion, we found that loss of Slap enhances the EPHB2 tyrosine phosphorylation (an index of EPHB2 activity) and association between EPHB2 and pSRC in isolated colonic epithelial cells, supporting increased signaling through this pathway (new Figure 4A). Furthermore, pharmacological inhibition of EPHB2 reduced both SRC activation and the hyperproliferative phenotype observed in Slap-deficient organoids (new Figure S2). Together, these findings provide functional and biochemical evidence that deregulated EPHB2 signaling contributes to SRC activation in the absence of SLAP. We also examined the effect of EPHB2 inhibition in human CRC tumoroids (new Figure 5).

Pharmacological EPHB2 inhibition reduced tumoroid growth in CRC cells expressing low levels of SLAP, whereas this effect was largely lost upon SLAP overexpression. Together, these new data identify EPHB2 as a critical upstream activator of SRC that is negatively regulated by SLAP and strengthen our conclusion that deregulated EPHB2-SRC signaling drives the hyperproliferative phenotype associated with SLAP loss.

To address the relevance of our findings in CRC patients, we also extended our analyses to human datasets (new Figure 5D). We observed a significant inverse correlation between SLAP expression and a colorectal cancer stem cell-like activity score in TCGA tumours. In addition, co-expression of SLAP and SLAP2 with EPHB2 was associated with improved disease-free survival in microsatellite-stable (MSS) CRC patients, whereas no such association was observed in microsatellite instability (MSI) tumours. These findings support the clinical relevance of the SLAP-EPHB2 signaling axis and are consistent with a role for SLAP in restraining EPHB2-dependent CSC signaling in CRC.

**Recommendations for the authors:**

**Reviewing Editor Comments:**

Both reviewers have reported that the study of the EPHB2-SLAP-Src axis is not very developed, as most results are derived from using inhibitors. A key question is whether EPHB2 is activated by SLAP depletion and whether it is critical to SRC activation. Addressing these questions would greatly improve the paper.

We thank the Reviewing Editor for this important suggestion. We would be happy for the editors to assess the revised version without involving the reviewers again. In the revised manuscript, we have substantially strengthened the mechanistic link between SLAP loss, EPHB2 activation, and SRC signaling. We show that Slap deletion increases both EPHB2 expression and tyrosine phosphorylation, enhances EPHB2-pSRC association in colonic epithelial cells. Importantly, pharmacological inhibition of EPHB2 suppresses SRC activation and rescues the hyperproliferative phenotype of Slap-deficient organoids. We further demonstrate that EPHB2 inhibition selectively impairs growth of CRC tumoroids with low SLAP expression, whereas this effect is largely abolished upon SLAP overexpression. An SH2- or SH3-inactivating point SLAP mutant failed to suppress tumoroid growth and restored sensitivity to EPHB2 inhibition, further supporting EPHB2 as a critical target of SLAP-mediated tumour suppression. Together, these new biochemical and functional data establish EPHB2 as a critical upstream activator of SRC that is negatively regulated by SLAP and significantly reinforce the central conclusions of the study.

**Reviewer #1 (Recommendations for the authors):**

(1) Evidence of SLAP expression in the colon is an important basis for these studies and could be moved to the main Figure 1 rather than being in the supplementary material.

We thank the reviewer for this suggestion. While we agree that documenting SLAP expression in the colon is important, we have retained these data in Figure S1 to maintain a concise main figure set, consistent with the recommended format for Short Reports.

(2) Define AOM/DSS and briefly describe the model at first mention. In addition, the model in 3A includes TAM treatment at 45 days, but this is not mentioned in the text. Why is this done?

We have better defined the AOM/DSS protocol at first mention in the revised manuscript and specified the rationale for tamoxifen administration at day 45, which is required to maintain efficient SLAP deletion throughout the duration of the experiment (90 days).

(3) Evidence that the SRC inhibitor decreased phospho-tyrosine levels in addition to inhibiting the growth of organoids should be included.

We included data showing the inhibitory effect of the used SRC inhibitor on global phospho-tyrosine levels in organoids in the revised manuscript.

(4) Further experiments investigating the involvement of EphB2 in colonic tumor formation are of interest and would increase the significance of this work.

We included data showing that SLAP modulation affects the response of tumoroids derived from cell lines to EphB2 inhibition, providing complementary mechanistic insights.

**Reviewer #2 (Recommendations for the authors):**

(1) The authors should confront their findings with data obtained in normal and pathological tissues: are SLAP, SRC, and EphB2 co-expressed, at the single cell level, in normal colon and CRC? In which cell populations? Is loss of SLAP associated with poor prognosis in CRC patients?

We thank the reviewer for this important suggestion. We agree that assessing the relevance of the SLAP-EPHB2-SRC axis in human CRC is important. However, transcriptomic datasets have inherent limitations in this context, as SLAP primarily regulates signaling at the post-transcriptional level and SRC activity cannot be reliably inferred from mRNA expression.

To address the clinical relevance of our findings, we performed additional analyses of CRC patient datasets. We found a significant inverse correlation between SLAP expression and a colorectal cancer stem cell-like activity score in TCGA tumours. Furthermore, co-expression of SLAP and SLAP2 with EPHB2 was associated with improved disease-free survival in microsatellite-stable (MSS) CRC patients. These findings are consistent with a role for SLAP in restraining EPHB2-dependent signaling in CRC. Finally, while our data identify EPHB2 as a critical upstream regulator of SRC signaling controlled by SLAP, we do not exclude the possibility that additional receptor tyrosine kinases contribute to the effects of SLAP loss during colorectal tumorigenesis.

(2) In Figure 4A, total EphB2 levels are increased in the absence of SLAP in colonic crypts. However, the level of EphB2 phosphorylation is not shown. This is an important point to address. Which ligand(s) may activate EphB2?

We agree that assessing EPHB2 activation is important. To address this point, we have included new data in the revised manuscript showing that Slap deletion increases EPHB2 tyrosine phosphorylation in isolated colonic epithelial cells, providing direct evidence that EPHB2 signaling is enhanced in the absence of SLAP. In addition, we show that loss of Slap increases EPHB2-pSRC association and that pharmacological inhibition of EPHB2 reduces SRC activation and suppresses the hyperproliferative phenotype of Slap-deficient organoids. Together, these findings establish EPHB2 as a critical upstream regulator of SRC signaling following SLAP loss.

Regarding EPHB2 activation, previous studies have shown that EPHB2 is primarily activated by ephrin-B ligands expressed within the intestinal crypt compartment (Batlle et al., 2002). EPHB2 signaling may also be reinforced through cooperation with other Eph receptors, particularly EPHB3, which is highly expressed in intestinal stem and progenitor cells (Holmberg et al., 2006; Genander et al., 2009). In addition, SRC has been reported to phosphorylate EPH receptors, raising the possibility of bidirectional signaling that could further amplify EPHB2-SRC pathway activity (Leroy et al., 2009; Hochgräfe et al., 2010).

(3) In the absence of SLAP, inhibitors of EphB2 should also decrease SRC activity as EphB2 lies upstream of SRC (Figure S3C). Does this occur in organoids?

We now show that EphB2 inhibition reduces SRC activity in SLAP-deficient organoids.

(4) What is the status of EphB2 and SRC (total, phosphorylated) in SW620 and HT29 CRC cells in the absence of SLAP?

SW620 and HT29 cells are SLAP-low CRC models. Given that SLAP expression is already minimal in these cells, further depletion is unlikely to provide meaningful additional insight.

(5) Expression of SLAP is associated with a decrease in the stem cell compartment in CRC cell lines (Figure S2). Is there a stem cell signature associated with low SLAP levels in CRC?

We analyzed TCGA colorectal cancer datasets using a published colorectal cancer stem cell (CSC) signature. We found a low but significant inverse correlation between SLAP expression and the CSC-like activity score, supporting our experimental observations that SLAP restrains stem cell properties in CRC cells and organoids.

(6) Does overexpression of the mutant form of SLAP (SLAPmut) limit SLAP effects in SW620 and HT29 CRC cells in Figure S2?

We have now performed the requested experiments and found that, unlike wild-type SLAP, SLAPmut failed to inhibit tumoroid growth in CRC cells. These results are consistent with our previous findings showing that SLAPmut lacks tumour suppressor activity in CRC cells (Naudin et al., Nat Commun, 2014) and further support the requirement of SLAP signaling functions for the regulation of CRC stem-like properties.

(7) Total SRC is missing in Figure 2B

Total SRC levels are now included in the revised figure.
